# Autophagy Protects the Blood-Brain Barrier Through Regulating the Dynamic of Claudin-5 in Short-Term Starvation

**DOI:** 10.3389/fphys.2019.00002

**Published:** 2019-01-18

**Authors:** Zhenguo Yang, Chunnian Huang, Yongfu Wu, Bing Chen, Wenqing Zhang, Jingjing Zhang

**Affiliations:** ^1^Department of Developmental Biology, School of Basic Medical Sciences, Southern Medical University, Guangzhou, China; ^2^Affiliated Hospital of Guangdong Medical University, Zhanjiang, China

**Keywords:** blood-brain barrier, claudin-5, permeability, starvation, autophagy

## Abstract

The blood-brain barrier (BBB) is essential for the exchange of nutrient and ions to maintain the homeostasis of central nervous system (CNS). BBB dysfunction is commonly associated with the disruption of endothelial tight junctions and excess permeability, which results in various CNS diseases. Therefore, maintaining the structural integrity and proper function of the BBB is essential for the homeostasis and physiological function of the CNS. Here, we showed that serum starvation disrupted the function of endothelial barrier as evidenced by decreased *trans*-endothelial electrical resistance, increased permeability, and redistribution of tight junction proteins such as Claudin-5 (Cldn5). Further analyses revealed that autophagy was activated and protected the integrity of endothelial barrier by scavenging ROS and inhibiting the redistribution of Cldn5 under starvation, as evidenced by accumulation of autophagic vacuoles and increased expression of LC3II/I, ATG5 and LAMP1. In addition, autophagosome was observed to package and eliminate the aggregated Cldn5 in cytosol as detected by immunoelectron microscopy (IEM) and stimulated emission depletion (STED) microscope. Moreover, Akt-mTOR-p70S6K pathway was found to be involved in the protective autophagy induced by starvation. Our data demonstrated that autophagy played an essential role in maintaining the integrity of endothelial barrier by regulating the localization of Cldn5 under starvation.

## Introduction

The blood-brain barrier (BBB) functions as a protective interface between the brain and the peripheral circulation. It serves to facilitate the influx and efflux of molecules and ions for maintaining the homeostasis of central nervous system (CNS) (Hawkins and Davis, 2005). BBB disruption is an essential early event in the pathogenesis of many neurological disorders including ischemia, neurodegenerative disease and brain infections, and is influenced by various physiological and pathological stimuli including nutrient status (Weiss et al., 2009). Endothelial cells of the cerebral capillary, which are connected by continuous intercellular tight junctions (TJs), are the main functional elements of the BBB in controlling cerebrovascular permeability (Carman et al., 2011). Claudins (Cldns), Occludin and Zonula Occludens (ZO) are main molecular components of TJs, protecting brain from damaging blood-borne agents (Hainsworth and Fisher, 2017). As a member of Cldn family, Cldn5 is highly expressed in the cerebrovascular endothelial cells to form cell-cell interaction and plays an essential role in sealing the paracellular clefts of adjacent cells (Stamatovic et al., 2011). Loss of Cldn5 or specific removal of Cldn5 from the membrane disrupted the permeability of BBB.

Autophagy is a conserved and survival process involved in intracellular degradation of misfolded proteins and cyclic utilization by lysosomes for maintaining cellular homeostasis (Gatica et al., 2015). Besides, it has also been demonstrated that autophagy contributes to clearance of apoptotic cells during embryonic development (Qu et al., 2007), and is associated with the pathophysiologies including degenerative diseases and inflammation (Deretic and Levine, 2017).

Defects of autophagy by mutation of autophagy-related genes (atgs) by inhibitors of autophagy could accelerate apoptotic process, which indicated that autophagy protects cells survival against apoptosis (Carpenter et al., 2008). Further study indicated that Akt-mTOR pathway was involved in autophagy in control of cell growth, differentiation and apoptosis (Saiki et al., 2011). When Akt-mTOR is inhibited by nutrients deprivation, autophagy is activated to maintain cell survival through Unc-51-Like Kinase 1 (ULK1) and Beclin-1 assisting phagophore assembling (Mizushima, 2010). Therefore, regulation of autophagy in a gentle condition may be benefit for the prevention and treatment of disorders.

Several recent studies also reported that autophagy could be rapidly activated by internal and external stimuli including nutrient deprivation (Mariño et al., 2014). This protective autophagy was stimulated to counteract apoptosis induced by starvation for maintaining intestinal barrier function (Nighot et al., 2015; Bai et al., 2017). When autophagy was inhibited, an impairment of barrier function was exacerbated as evidenced by decreased expression of TJ proteins Cldn1 and ZO-1 (Yang et al., 2015). However, adequate evidence is still lacking to elucidate the role of autophagy in the maintenance or disruption of BBB.

In this study, autophagy and its effect on the integrity of BBB were investigated in cerebral endothelial cells fed with 2% serum to mimic nutrient starvation. The protective role of autophagy was revealed to elevate the *trans*-endothelial electrical resistance (TEER) and lower the paracellular permeability by inhibiting the redistribution of membrane Cldn5 through ROS scavenging, in which Akt-mTOR-p70S6K pathway was suppressed.

## Materials and Methods

### Cell Culture and Treatment

Mouse brain microvascular endothelial cells (bEnd.3, Bioleaf Biotech, China) were maintained in Dulbecco’s modified Eagle’s medium (DMEM, Life, United States) supplemented with 10% fetal bovine serum (FBS, Hyclone, United States) at 37°C in a 5% CO_2_ incubator. Human cerebral microvascular endothelial cells (hCMEC/D3, Bioleaf Biotech, China) were cultured in EBM-2 basal medium and EGM-2^®^-MV SingleQuots^®^ kit (including FBS, hydrocortisone, human fibroblast growth factor-B, human vascular endothelial growth factor, insulin-like growth gactor-1, ascorbic acid, gentamicin, amphotericin B) from Lonza (Basel, Switzerland). After a confluent monolayer formation, the starvation experiment was performed as previously reported with slight modifications (Shaked et al., 2005). In brief, the bEnd.3 or hCMEC/D3 monolayer cells were cultured in medium supplemented with 2% FBS as starvation group or with 10% FBS as control group for the indicated time periods. For autophagy analyses, medium with 10% FBS, 50 nmol/L rapamycin (Rapa, Cell Signaling Technology, United States), 30 μmol/L chloroquine (CQ, Sigma-Aldrich, United States) or 10 mmol/L 3-methyladenine (3-MA, Sigma-Aldrich, United States) was applied to incubate the monolayer cells for 2 h and then with 2% FBS containing consistent concentrations of Rapa, CQ and 3-MA respectively.

### Measurement of TEER and Paracellular Permeability

The TEER value was measured to reflect the barrier property of the bEnd.3 monolayer as previously described (Liao et al., 2016). Briefly, bEnd.3 cells were seeded at a density of 3 × 10^4^ cells/cm^2^ on collagen-coated semi permeable membrane filters with a surface area of 0.33 cm^2^ and 0.4 μm pore size (Millipore, United States). After incubation in a 5% CO_2_ incubator for 24 h, the resistance of inserts was monitored each 4 h by CellZscope^®^-System (nanoAnalytics GmbH, Muenster, Germany) prior to and during followed by 80 h of starvation treatment. TEER value of the inserts was measured for cell layers by subtracting the resistance of control inserts. Data was analyzed and presented as percentage of TEER.

Besides the measurements of TEER, paracellular permeability of endothelial cell monolayer (bEnd.3 or hCMEC/D3) was determined using FITC-labeled dextran (10 kDa, 0.5 mg/L, Thermo-Fisher, United States). For the measurement of paracellular permeability, bEnd.3 and hCMEC/D3 were seeded on semi permeable membrane filters respectively. After growth for 5 days, endothelial cell monolayers were incubated with FITC-dextran in HBSS buffer for 60 min at 37°C in incubator with 5% CO_2_. The lower chamber was chosen and the fluorescence was measured with a spectrophotometer-computer interfaced system (BioTek Epoch, United States) at a wave length of 594 nm. Apparent permeability coefficient (Papp) was calculated as previously reported (Gaillard et al., 2001). For the measurements of TEER and permeation of tracers, 9 filters of bEnd.3 or hCMEC/D3 cell lines were investigated for each group.

### Detection of Apoptosis

To quantify apoptosis, cells were harvested and stained with annexin V-FITC and propidium iodide (Roche, United States). The apoptotic cells (annexin V-positive and PI-negative) were analyzed using a FACSCalibur flow cytometer (BD Biosciences, United States).

### Immunofluorescence Staining and Immunoblotting

The localization of TJ proteins and LC3 was assessed by immunofluorescence staining. Monolayers of bEnd.3 or hCMEC/D3 were fixed with acetone for 10 min on ice and blocked (1% bovine serum albumin and 0.2% Tween 20 in PBS) for 1 h. The fixed cells were then incubated with primary antibodies, including anti-ZO-1 (Invitrogen, 33-9100), anti-Cldn5 (Invitrogen, 35-2500), anti-LC3 (CST, 4108), diluted 1:200 in blocking solution overnight at 4°C and subsequently incubated with Alex Fluro 488-conjugated goat anti-mouse IgG, Alex Fluro 647-conjugated goat anti-rabbit IgG (1:200; Jackson ImmunoResearch, United States) and DAPI (1:1000; Sigma-Aldrich, United States) for 1 h at 37°C. Finally, the cells were imaged using a Leica TCS SPII 5 confocal microscope (Leica, Solms, Germany). To analyze the degradation of Cldn5 after starvation, the localization of Cldn5 in autophagosome was imaged by Leica TCSSP8 STED 3X microscope (Leica, Solms, Germany).

For immunoblotting, cells were harvested and lysed on ice with RIPA buffer kit (Beyotime, China) including protease inhibitor complex (Beibo, China) and then centrifuged at 12,000 *g* for 10 min at 4°C. After centrifugation, the cell lysates were collected and the protein concentration was measured. Equal amounts of denatured protein (40 ng per lane) were separated by sodium dodecyl sulfate polyacrylamide gel electrophoresis (SDS-PAGE), and then transferred to PDVF membranes (Millipore, United States). After incubation in blocking solution (5% skimmed milk) for 1 h at room temperature, the membranes were incubated with primary antibodies, including anti-LC3 (CST, 4108), anti-ATG5 (CST, 12994), anti-mTOR (CST, 2983), anti-p-mTOR (CST, 5536), anti-LAMP1 (Santa Cruz, AB24170), anti-Akt (Proteintech, 60203-2-Ig), anti-p-Akt (Proteintech, 66444-1-Ig), anti-p70S6K (CST, 2708), anti-p-p70S6K (CST, 9234), diluted 1:1000 and horseradish peroxidase-conjugated secondary antibodies successively. Subsequently, western blot bands were observed with ECL advance western blotting detection reagents (Millipore, United States) and imaged by Bio-Rad ChemiDoc^TM^ MP imaging system (Bio-Rad Laboratories, Hercules, CA, United States).

### Transmission Electron Microscopy (TEM)

Cells for electronic microscopy were prepared as previous described (Jiang et al., 2016). In brief, samples were fixed with 2.5% glutaraldehyde and 1% osmium tetroxide for 12 h. After washed with PBS, samples were dehydrated in graded ethanol and embedded in plastics. The sections were then prepared and stained with uranyl acetate and lead citrate. Representative areas from the sections were viewed with a JEM-1400 electron microscope (JEM, Tokyo, Japan), and the autophagic vacuoles from the whole cell were quantified.

To determine the autophagosome-like vesicles and the subcellular localization of Cldn5 in bEnd.3 cells after serum starvation, immunoelectron microscopy (IEM) was performed as previously described with slight modifications (Rivassantiago et al., 2005). Briefly, cells were fixed for 4 h with 4% paraformaldehyde and 2% glutaraldehyde in 0.2 mol/L sodium phosphate buffer. After that, cells were dehydrated in increasing concentrations of alcohol, and infiltrated with increasing concentrations of LR-White resin (London Resin, United Kingdom) on ice. Sections were cut at 70–80 nm thick and placed on nickel grids. Then, the nickel grids were incubated with monoclonal mouse anti-Cldn5 (1:50; Invitrogen, United States) overnight at 4°C, and subsequently incubated with goat anti-mouse IgG conjugated to 10-nm gold particles (Sigma-Aldrich, United States) for 2 h at room temperature. Finally, the gold labels were imaged by JEM-1400 electron microscope.

### Detection of Reactive Oxygen Species (ROS)

The ROS levels in bEnd.3 cells were determined using ROS assay kit (Beyotime, China) according to the manufacturer’s instructions. Briefly, the bEnd.3 monolayer on cover slips for ROS detection was incubated with 2′,7′-Dichlorodihydrofluorescein diacetate (DCFH-DA, 10 μmol/L) in serum-free medium at 37°C in a 5% CO_2_ incubator for 20 min. Thereafter, the cells were washed in PBS for three times and the fluorescence was examined by a Leica TCS SPII 5 confocal microscope.

### Statistical Analysis

In this study, all experiments were presented as means ± standard error (mean ± SEM). Two-tailed Student’s *t*-test was performed to test for significant differences between two groups. For multiple groups, the comparisons were made by one-way analysis of variance (ANOVA) and the Least Significant Difference *post hoc* test. *P* < 0.05 and *P* < 0.01 were indicated by ^∗^ and ^∗∗^ respectively.

## Results

### Starvation Impairs the Permeability of Brain Endothelial Barrier

**FIGURE 1:**
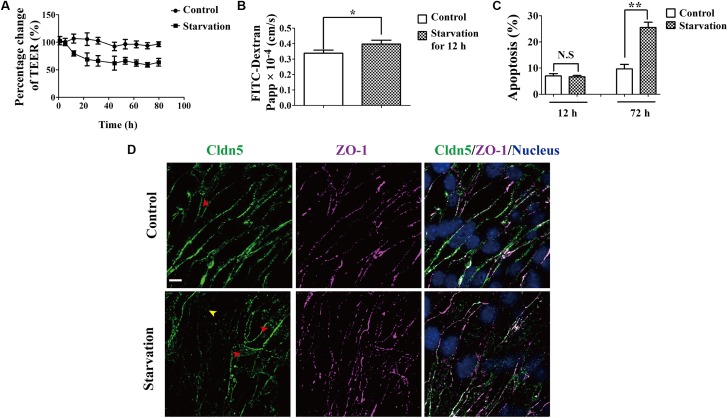
Effect of serum starvation on the integrity of brain endothelial cell barrier. bEnd.3 cells were exposed to control medium (10% FBS) or serum starvation medium (2% FBS) after the formation of tight cell monolayers. **(A)** TEER values were analyzed and presented as percentage of TEER at indicated time points (1, 5.3, 12, 22.5, 31.5, 44.5, 53, 62, 71, 80 h). **(B)** Paracellular permeability was explored for 12 h. **(C)** Apoptosis of endothelial cell barrier after exposure to vehicle and starvation. **(D)** Localization of Cldn5 and ZO-1 was measured by immunofluorescence staining. Representative data from three separate experiments were shown here, ^∗^*P* < 0.05, ^∗∗^*P* < 0.01. Cldn5 in membrane (yellow arrow head) or cytosol (red arrow heads) indicated its redistribution after starvation. Papp, apparent permeability coefficient. Scale bar: 10 μm.

To evaluate the effect of starvation on the BBB, TEER values were measured on cell culture insert, where bEnd.3 cells grew and were incubated with 2% FBS for serum starvation. TEER values from starvation group decreased in a time-dependent manner (Figure 1A). It dropped rapidly after starvation treatment and showed a significant difference comparing with that of the control group from about 4–80 h. The TEER reached a stable value at 24 h post treatment. In parallel, the flux of FITC-conjugated dextran across the bEnd.3 monolayer was also measured to reflect the paracellular permeability. The permeability increased dramatically after starvation treatment for 12 h, in comparison with the control group (0.40 ± 0.07 × 10^-4^cm/s and 0.33 ± 0.07 × 10^-4^ cm/s; starvation *vs.* control *P* < 0.01, Figure 1B). This is consistent with the TEER changes of bEnd.3 monolayer under starvation. In addition, we found that the starvation treatment could not induce marked apoptosis in endothelial cells (Figure 1C).

Since endothelial TJs are essential structure for maintaining the BBB function, the expression and localization of Cldn5 and ZO-1 in bEnd.3 were investigated by immunofluorescent staining. As shown in Figure 1D, Cldn5 was partly lost from cell-cell contacts (yellow arrow head) and appeared to cytosolically aggregated post starvation for 12 h (red arrow heads). While ZO-1 still localized on cell-cell contacts. These results suggested that the endothelial barrier dysfunction induced by starvation might be due to the redistribution Cldn5 in endothelial cells.

### Autophagy Is Activated in Cerebral Endothelial Cells After Starvation

**FIGURE 2:**
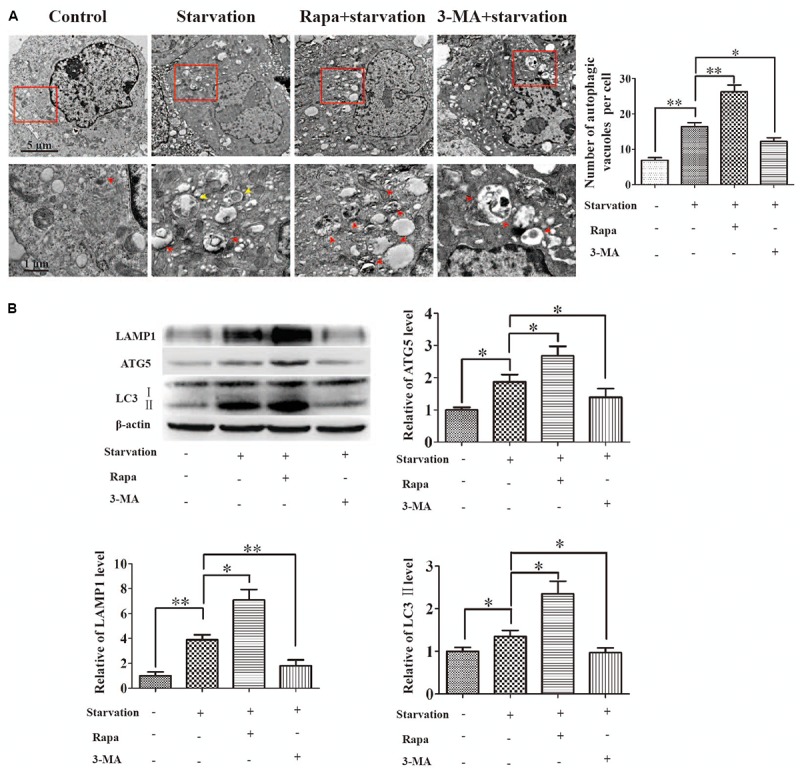
Autophagy was activated by starvation in brain endothelial cells. 3-methyladenine (3-MA) or rapamycin (Rapa) in medium with 10% FBS were applied to pretreat for 2 h in bEnd.3 monolayer cells, and during the following serum starvation experiments. **(A)** Autophagic vacuoles were observed by TEM. Autophagosomes (red and yellow arrow heads) in the red boxed areas were magnified. Partial mitochondria (yellow arrow heads) were fused by autophagosomes in starvation group. **(B)** Protein levels of LAMP1, ATG5, and the ratio of LC3II/I were analyzed by western blot for 12 h starvation. Data shown were mean ± SEM for four independent experiments. ^∗^*P* < 0.05, ^∗∗^*P* < 0.01, comparing with the corresponding control group.

It has been reported that autophagy could be induced after nutrient deprivation stimulus (Geeraert et al., 2010). We therefore asked whether autophagy could be activated in brain endothelial cells under serum starvation. To this end, bEnd.3 cells after starvation treatment were analyzed by TEM and the autophagic vacuoles were visualized (Figure 2A). The number of autophagic vacuoles in starvation-treated cells was apparently increased in comparison with that of the cells cultured with normal serum. Rapa is widely used as a novel enhancer of autophagy to relieve the inhibition of ULK1 and assist the formation of autophagosome through inhibiting phosphorylation of mTOR (Nazio et al., 2013), while 3-MA, as a class III PI3K inhibitor, could prevent autophagosome formation (Seglen and Gordon, 1982). Therefore, the formation of autophagic vacuoles in cerebral endothelial cells incubated with Rapa or 3-MA under starvation was analyzed in parallel to further prove the specific induction of autophagy in bEnd.3 cells by serum starvation. As a result, the autophagic vacuole number was significant increased in Rapa-treated group comparing with that in starvation group (Figure 2A). In contrast, the accumulation of autophagic vacuoles was abolished in 3-MA-treated group (Figure 2A). In addition, we also evaluated the autophagic flux by analyzing the expression of proteins associated with autophagy, such as LAMP1, AGT5 and LC3 (Figure 2B). Expression levels of LC3II/I, LAMP1 and AGT5 in bEnd.3 cells were remarkably increased under starvation. Their expression was further increased by Rapa treatment, but efficiently reduced by 3-MA incubation. These outcomes indicated that serum starvation treatment activated autophagy in endothelial cells.

It has been reported that Akt-mTOR-p70S6K pathway is involved in the regulation of nutrition metabolism and autophagy could be activated by suppressing Akt-mTOR-p70S6K axis (Shinji et al., 2011). Therefore, we further investigated whether Akt-mTOR-p70S6K axis was involved in the starvation-induced autophagy in bEnd.3 cells. To this end, the temporal expression of p-mTOR (S2448), p-Akt (Ser473), and p-p70S6K (Thr389) proteins were analyzed in bEnd.3 cells after starvation for 12 h. As shown in Supplementary Figure S1, with the increase of LC3II/I in a time-dependent manner under starvation, the expression of p-mTOR, p-Akt and p-p70S6K was inhibited. This data indicated that starvation induced autophagy *via* Akt-mTOR-p70S6K inhibition in endothelial cells.

### Autophagy Protects Endothelial Barrier From Disruption Under Starvation

**FIGURE 3:**
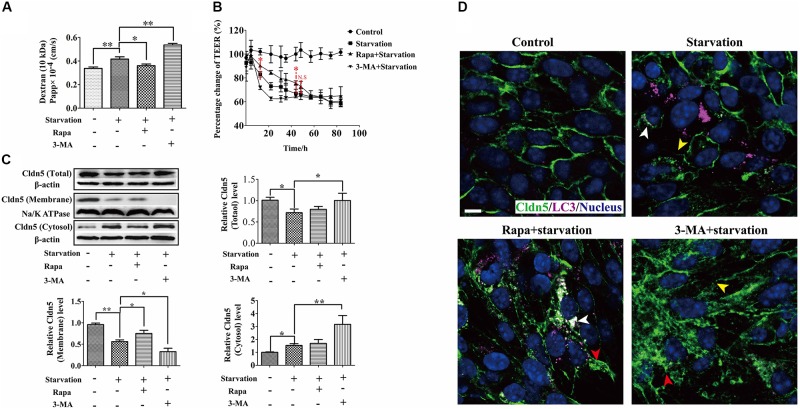
Autophagy protected integrity of brain endothelial cell barrier from the impairment of serum starvation through regulating the dynamic of Cldn5. **(A,B)** The role of autophagy on the property of endothelial cell barrier was measured by TEER and paracellular permeability. **(C)** Cldn5 protein in the total cell extract, membrane fraction, and cytosol fraction was measured by western blot analysis. **(D)** The localization of Cldn5 (Green) and LC3 (Purple) was analyzed by immunofluorescent staining. Cldn5 lost from cell-cell contacts (yellow arrow heads) and aggregated in cytosol (red arrow heads). The aggregated Cldn5 was degraded by autophagy (white arrow heads). 3-MA, 3-methyladenine; Rapa, rapamycin. Data are provided as the mean ± SEM. ^∗^*P* < 0.05, ^∗∗^*P* < 0.01, comparing with the control group. Scale bar: 10 μm.

Autophagy has been demonstrated to maintain the integrity of microvascular barrier including BBB (Li et al., 2014), glomerular barrier (Yi et al., 2017; Matsuda et al., 2018), and alveolar barriers (Dong et al., 2018). Therefore, we next investigated the effects of autophagy on the endothelial barrier function by determining TEER and paracellular permeability. As a result, TEER measurement showed a further decrease of trans-cellular resistance after the abrogation of autophagy by 3-MA treatment from 12.5 to 30.8 h, and a significant increase by Rapa treatment from 12.5 to 48.4 h comparing with that in starvation group (Figure 3A). Consistently, the paracellular permeability increased after starvation for 12 h. This leakiness was inhibited by Rapa treatment, while was enhanced by 3-MA treatment (Figure 3B). The change of TEER and the paracellular permeability indicated that autophagy could partly ameliorate the breakdown of cerebral endothelial barrier induced by starvation. To further prove this finding, we also used hCMEC/D3 to analyze the activation of autophagy under starvation and its effects on the permeability of hCMEC/D3 cell monolayer. As a result, starvation treatment for 12 h activated autophagy in hCMEC/D3 cells and caused an increased permeability of cell monolayer, as well as in bEnd.3 cells (Supplementary Figure S2A). Meanwhile, Rapa treatment preserved the tightness of cell monolayer. In contrast, 3-MA treatment exacerbated breakdown of the tightness of endothelial barrier (Supplementary Figure S2B). Based on the above evidence that autophagy could protect endothelial cell barrier from breakdown during early starvation, we next asked for the molecular mechanism involved in this protective process.

### Autophagy Protects Endothelial Barrier *via* Affecting the Dynamic of Cldn5 Under Starvation

As one of trans-membranous TJ proteins, Cldn5 seals the paracellular space of endothelial cells by its *trans*-/*cis*- interactions to form cell-cell contacts and TJ strands (Piontek et al., 2008). Our previous study has shown that Cldn5 on the membrane of cell-cell contacts is necessary for the BBB function (Liao et al., 2016). Therefore, the expression and localization of endothelial Cldn5 under starvation were evaluated firstly by immunoblotting and immunofluorescent staining. As shown in Figure 3C, comparing with the control group, the total and membrane fraction of Cldn5 reduced, but the amount of cytosolic Cldn5 increased in starvation-induced bEnd.3 cells. Rapa treatment could partially inhibit the loss of membrane Cldn5, while 3-MA treatment increased the cytosolic fraction of Cldn5. Consistently, the immunocytochemical studies confirmed the loss of membrane Cldn5 (yellow arrow heads in Figure 3D) and accumulation of Cldn5 in cytosol in early starvation (red arrow heads in Figure 3D). Interestingly, the redistribution of membrane Cldn5 was inhibited after Rapa treatment as shown by the amounts of Cldn5 clusters on cell membrane, and 3-MA treatment enhanced the redistribution of Cldn5 in cytosol. These results indicated that the starvation-induced impairment of BBB function might be related to the redistribution of membrane Cldn5, and autophagy played a protective role in maintaining BBB integrity through inhibiting the cellular redistribution of Cldn5.

**FIGURE 4:**
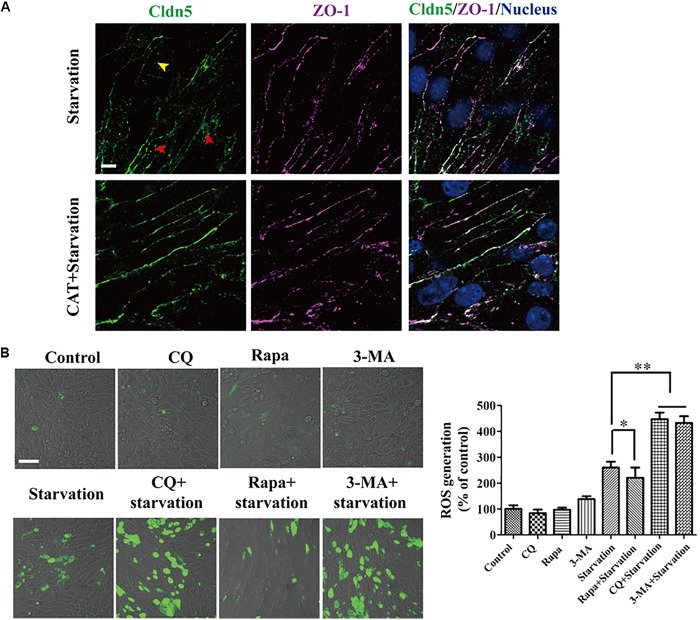
ROS generation was induced by serum starvation and autophagy was available for ROS scavenging. **(A)** The redistribution of Cldn5 from the membrane fraction (yellow arrow head) to cytosol (red arrow heads) was suppressed in bEnd.3 monolayer after incubated in medium with 2% FBS and CAT (2000 U/mL) for 12 h. Scale bar: 10 μm. **(B)** ROS in bEnd.3 cells indicated by DCF fluorescence (green) was imaged by confocal microscopy. The relative level of ROS generation was quantified and was reduced by autophagy. Representative images and quantifications of cells with DCF fluorescence are presented. ^∗^*P* < 0.05, ^∗∗^*P* < 0.01. 3-MA, 3-methyladenine; Rapa, rapamycin; CQ, chloroquine. Scale bar: 50 μm.

As reactive oxygen species (ROS) was reported to induce the degradation of TJ proteins and therefore resulted in BBB dysfunction (Gangwar et al., 2017), we next analyzed the role of ROS on the localization of TJ components and explored the effect of autophagy on ROS scavenging. First, ROS was confirmed to be caused in bEnd.3 cells by starvation (Figure 4B) and scavenging of ROS by catalase (CAT) could efficiently inhibit the delocalization of Cldn5 from membrane (Figure 4A). Activating the autophagy by Rapa could efficiently block the production of ROS, while interfering the autophagy pathway by CQ or 3-MA led to an increase of intracellular ROS levels (Figure 4B). Since it was evidenced that ROS was generated by dysfunctional mitochondria (Indo et al., 2007), we also observed autophagy-mediated degradation of damaged mitochondria under starvation by TEM analyses (Figure 2A, yellow arrow heads), which may reduce the production of ROS. Above findings proved that autophagy protected cerebral endothelial barrier through inhibiting the ROS production in serum starvation.

### Autophagy Mediates the Clearance of Aggregated Cldn5 in Endothelial Cells Under Starvation

**FIGURE 5:**
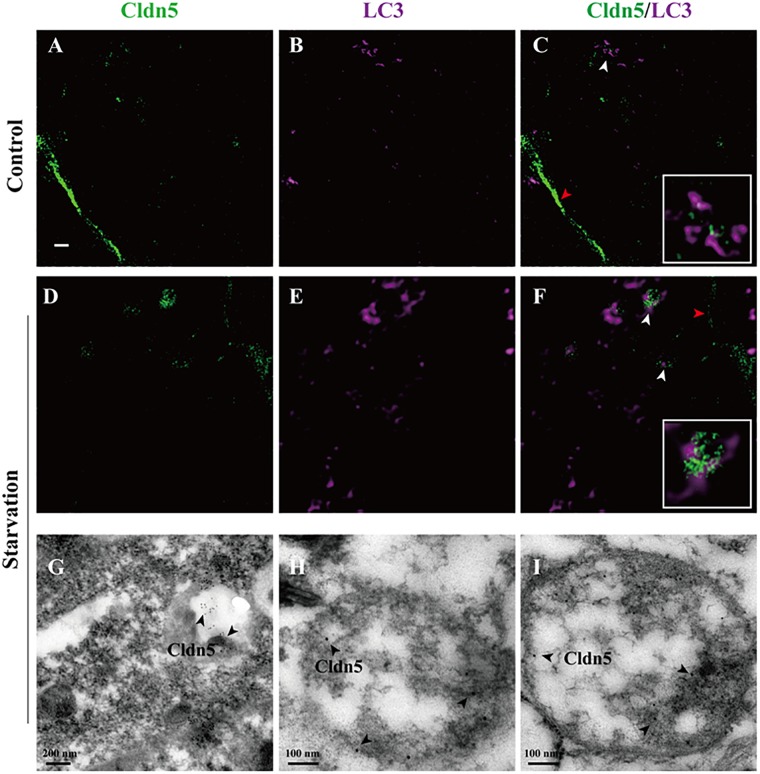
Proximity of autophagosomes and Cldn5 in endothelial cells. **(A–F)** STED Microscopy imaged the localization of LC3 (purple) and Cldn5 (green) in bEnd.3 cell. Starvation caused a reduced membrane localization of Cldn5 (red arrow heads) and led to accumulation of Cldn5 which was packaged by autophagosomes in cytosol (white arrow heads, enlarged in white frame). Scale bar: 1 μm. **(G–I)** Immunogold-labeled Cldn5 was evident within autophagosome (black arrow heads).

Abnormal protein aggregation has been reported to cause neurodegenerative diseases and aging, such as Alzheimer disease (Martínez et al., 2010). Autophagy in endothelial cells is critical for maintaining cell survival by eliminating abnormal or misfolded intracellular proteins (Quinones et al., 2012). Our results demonstrated that aggregated Cldn5 in cytoplasm mainly co-localized with LC3 in starvation-induced endothelial cell (white arrow heads in Figure 3D and Supplementary Figure S2A), indicating a role of autophagy on the clearance of aggregated Cldn5. To prove this, STED microscopy was first applied to assess the localization of Cldn5 and autophagosome. The results showed that cytosolic Cldn5 was packaged in autophagosome especially in starvation group (Figures 5A–F). Immunogold labeling TEM was then performed to further confirm this finding. As a result, the immunogold-labeled Cldn5 was detected in the mature autophagosome-like vesicles in starvation group (Figures 5G–I). These findings elucidated that the accumulated Cldn5 in cytosol was eliminated by autophagosome to prevent further cell damage under starvation.

## Discussion

In the present study, we demonstrated that endothelial barrier was impaired due to a redistribution of Cldn5 from cell membrane into the cytosol after serum starvation treatment. Meanwhile, autophagy was activated and showed a protective role in maintaining the integrity of endothelial barrier through facilitating the removal of aggregated Cldn5 and excess ROS in cytosol induced by serum starvation.

Blood-brain barrier disruption in neurological disorders such as infectious, ischemic stroke, and degenerative diseases, is considered to aggravate the pathogenesis and progression of neuronal networks dysfunction and degeneration (Zhao et al., 2015). In our study, the dysfunction of endothelial barrier was induced by serum starvation, as evidenced by the decrease of TEER and increase of paracellular permeability. This is in agreement with the previous study showing that the intestinal barrier using epithelial cells was impaired by nutrients deprivation, as evidenced by TEER decrease, permeability enhancement and decreased expression of TJ proteins including Cldn1 and ZO-1 (Yang et al., 2015). Since BBB properties are mainly dependent on the cerebral endothelial layer of the capillaries and the TJ complex at the most apical region of the paracellular space between adjacent endothelial cells, the integrity of TJs, especially the localization of endothelial Cldn5, was further explored in our study. Immunocytochemical studies showed a redistribution of Cldn5 from cell membrane into the cytosol after starvation treatment (Figures 1D, 3D). This was directly related with the change of TEER and endothelial permeability (Figures 1A,D and Supplementary Figure S2). It has been proved that the translocation of junction proteins including VE-Cadherin, ZO-1 and Occludin induced by oxygen/glucose deprivation was the main cause in impairment of BBB (Shi et al., 2017). All together, these results indicated that endothelial Cldn5 might be the potential TJ component affected by serum starvation.

Autophagy is a conserved cellular event relying on lysosome for the degradation of misfolded proteins and dysfunctional organelles, and providing necessary energy and amino acids for cell survival against intracellular or extracellular stimuli including starvation. The autophagic elimination of abnormal proteins is also considered as a way of cellular protection to avoid the cellular toxicity of aggregated protein (Ishida et al., 2009). However, the detailed mechanism of autophagy in the context of cellular survival and disease is still not well understood. Here, we showed that autophagy was activated as observed by autophagosome formation and the up-regulation of autophagic markers, such as LC3, ATG5 and LAMP1 under starvation (Levine and Kroemer, 2008). When autophagy was accelerated, the tendency of the decrease of TEER or the increase of paracellular permeability was inhibited, suggesting a protective role of autophagy in the impairment of endothelial barrier under early serum starvation. This protective effect could be enhanced or inhibited when accompanying with the autophagy activator- or inhibitor- treatment (Figures 3A,B). However, after a long term starvation (48.4 h), no protective or damage effect of autophagy was detected after 48.4 h treatment (Figure 3B). This might be due to a caspase-dependent apoptosis, instead of autophagy, resulting in endothelial barrier impairment after long term of starvation treatment (Figure 1C). When imaged by IEM and STED microscope, the aggregated Cldn5 in cytosol induced by starvation was found to be packaged by autophagosome (Figure 5). Subcellular fraction of Cldn5 determined by immunoblotting indicated that autophagy protected the integrity of endothelial barrier from breakdown through inhibiting the redistribution of delocalized Cldn5. Inhibition of autophagy by 3-MA aggravated the accumulation of Cldn5 in cytosol (Figure 3C), demonstrating that autophagy might be involved in mediating the degradation of the aggravated Cldn5 induced by starvation. Timely and effective removal of abnormally aggregated proteins is conducive to cell survival (Hisham et al., 2015). However, in our study, we found that the enhancement of autophagy by Rapa did not aggravate the decline level of total Cldn5 (Figure 3C). This might be due to the new synthesized Cldn5 in endothelial cells. Previous studies have demonstrated that Cldn5 degradation in bEnd.3 cells was mediated by autophagy dependent of caveolin-1 under oxygen-glucose deprivation (OGD) for 4 h, while only Cldn5 redistribution not degradation for 2-h OGD (Liu et al., 2015). However, our results showed that both Cldn5 redistribution and degradation in endothelial bEnd.3 cells were affected followed by serum starvation treatment with 2% FBS for 12 h. The explanation might be that the status of Cldn5 (redistribution or degradation) is dependent on the types of stimulus and its processing time. A short term (12–48 h) of starvation-induced autophagy in this study is demonstrated to show protective effect on endothelial barrier integrity through clearance of the aggregated Cldn5 (Figures 3B, 5). However, A long-term treatment of starvation (around 52–80 h) might result in the degradation of key proteins of cell metabolism, which accelerates the starvation-induced apoptosis.

The protective role of autophagy was further confirmed by ROS clearance, therefore avoiding the redistribution of endothelial membrane Cldn5 and the impairment of endothelial barrier. Previous studies demonstrated that ROS induced autophagy is related with the mitochondrial dysfunction in diverse pathological conditions including nutrient starvation (Scherz-Shouval and Elazar, 2011). In reverse, ROS could also be removed by autophagy (Zhang et al., 2016). Recently, Matsuda et al. (2018) has shown that autophagy protects the integrity of glomerular capillaries against ROS attack. In this study, we demonstrated that autophagy could mediate ROS scavenging. This indicated that autophagy played a protective role as an anti-oxidant system in the clearance of ROS to avoid endothelial TJ damage. It is of note that ROS generation induced by starvation did not resulted in cell apoptosis in our study (Figures 1C, 4B). The underlying reason could be that ROS may not be necessarily associated with apoptosis. This explanation could also be supported by the finding that mitochondrial ROS formation does not undergo apoptotic cell death in fission yeast (Hickson-Bick et al., 2002).

What’s more, autophagy induced by starvation in this study was proved *via* the inhibition of Akt-mTOR-p70S6K. Previous study has reported that Akt-mTOR signaling integrates external stimuli including nutrient deprivation to serve as a central regulator of cell growth and survival (Kim et al., 2015). Akt-mTOR-p70S6K pathway is demonstrated to be involved in induction of autophagy (Saiki et al., 2011). When the external nutrient is limited, phosphorylations of Akt, mTOR and p70S6K are thus inhibited to activate autophagy, therefore providing energy and amino acids for cell survival against nutrient limitation conditions. In our study, we showed that autophagy was enhanced by Rapa, a negative regulator of mTOR (Figures 2A, 3D). Moreover, starvation inhibited the phosphorylation of Akt, mTOR and p70S6K, which was correlated with the increased expression of LC3II/I in a time-dependent manner. These results indicated that Akt-mTOR-p70S6K signaling was involved in autophagy under starvation.

In summary, our study revealed that serum starvation induced an impairment of endothelial barrier, which was correlated with the redistribution of the tight junction proteins especially Cldn5 from endothelial cell membrane into the cytosol. Meanwhile, autophagy was activated *via* Akt-mTOR-p70S6K inhibition, and could protect the integrity of brain endothelial barrier from breakdown during the early starvation induction. Cldn5 was shown as a potential target of autophagy and the redistribution of Cldn5 was mediated by autophagy through ROS scavenging under starvation.

## Author Contributions

ZY designed and performed the experiments, analyzed the data, and wrote the manuscript. CH and YW performed the experiments and analyzed the data. BC and WZ reviewed the data and gave extract suggestions on this study. JZ initiated the study, designed the experiments, analyzed the data, and wrote the manuscript.

## Conflict of Interest Statement

The authors declare that the research was conducted in the absence of any commercial or financial relationships that could be construed as a potential conflict of interest.
