# Supplementary figures and images for: Autophagy Protects the Blood-Brain Barrier Through Regulating the Dynamic of Claudin-5 in Short-Term Starvation
Source: Front Physiol. 2019 Jan 18;10:2. doi: 10.3389/fphys.2019.00002 (PMC6345697; doi:10.3389/fphys.2019.00002)

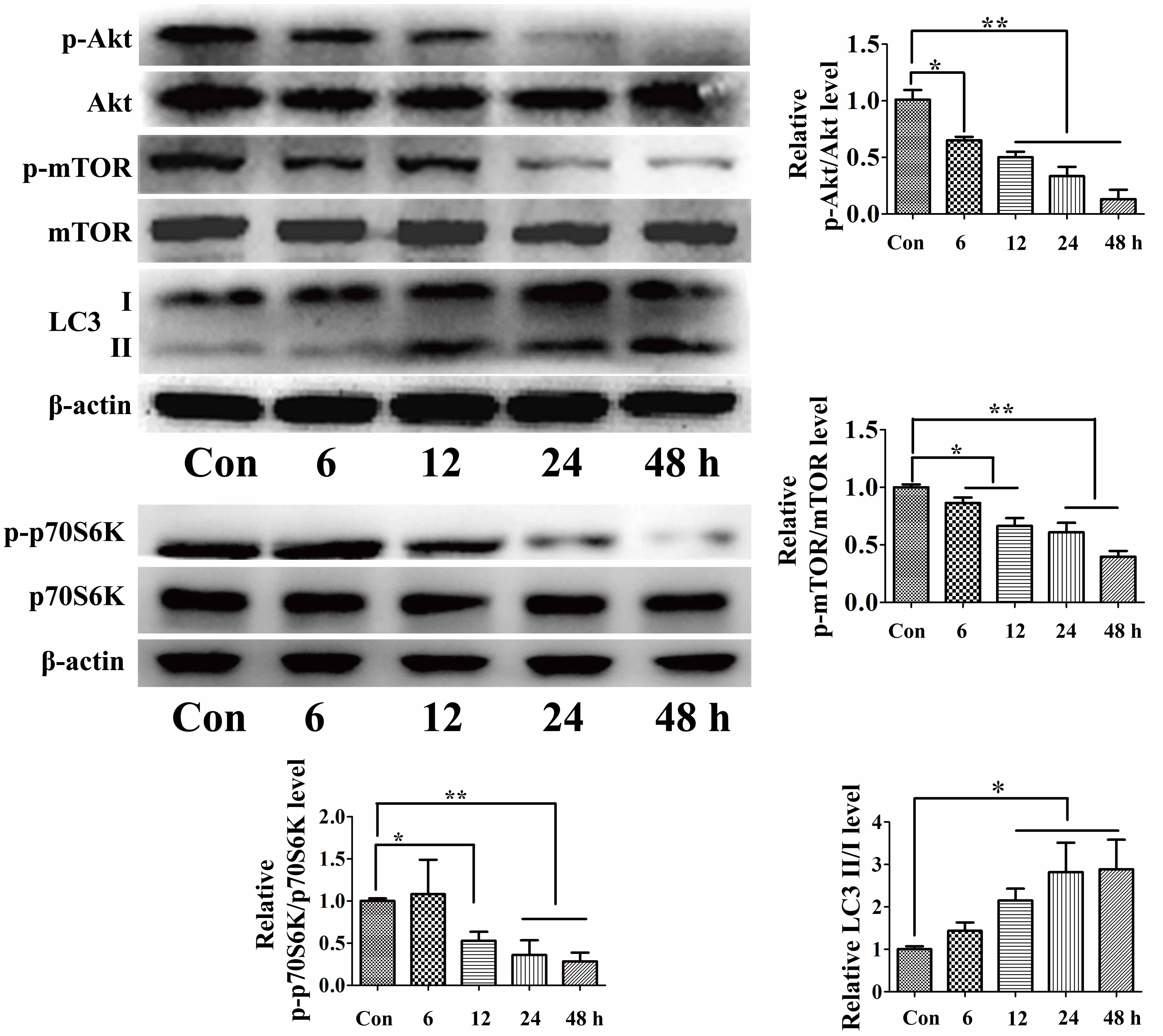

Supplement: Supplementary file 1 [file Image_1.jpeg]

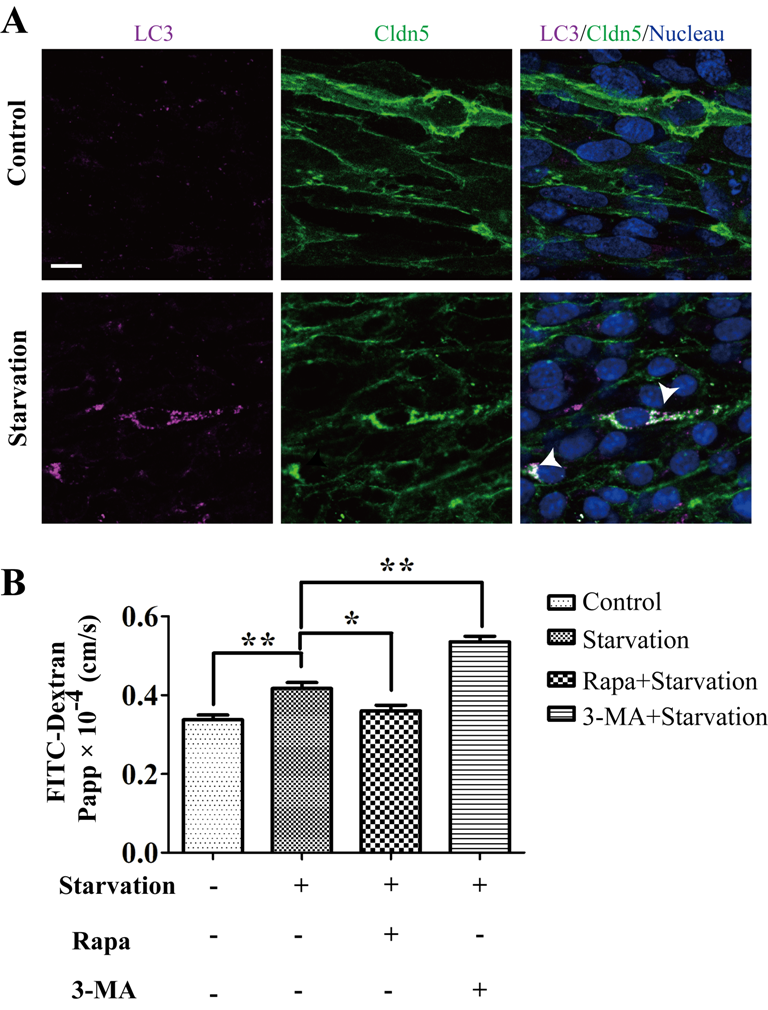

Supplement: Supplementary file 2 [file Image_2.tif]
