# Supplementary material for: Autophagy Protects the Blood-Brain Barrier Through Regulating the Dynamic of Claudin-5 in Short-Term Starvation
Source: Front Physiol. 2019 Jan 18;10:2. doi: 10.3389/fphys.2019.00002 (PMC6345697; doi:10.3389/fphys.2019.00002)
Supplement: Supplementary file 3 [file Data_Sheet_1.pdf]

Supplementary information

FIGURE LEGENDS

FIGURE S1 | Akt-mTOR pathway was involved in activation of autophagy induced by serum starvation. Western blot was used to determine the relative expression of p-Akt, p-mTOR, LC3-II/I and p-p70S6K in bEnd.3 monolayer under starvation for the indicated time. Data are provided as the mean ± SEM. * $P < 0.05$, ** $P < 0.01$, comparing with the control group.

FIGURE S2 | **Autophagy was activated under starvation and played a protective role in the integrity of endothelial cell barrier formed by hCMEC/D3.** (A) Localization of LC3 and Cldn5 was measured by immunofluorescence staining. Cldn5 aggregated in cytosol and autophagy mediated its clearance (white arrow heads). (B) Autophagy played a role in reducing the paracellular permeability under starvation. 3-MA, 3-methyladenine; Rapa, rapamycin. Data are provided as the mean ± SEM. * $P < 0.05$, ** $P < 0.01$. Scale bar: 10 µm.
